# Polyurethane/Zinc Oxide (PU/ZnO) Composite—Synthesis, Protective Property and Application

**DOI:** 10.3390/polym12071535

**Published:** 2020-07-11

**Authors:** Mohammad Mizanur Rahman

**Affiliations:** Center of Research Excellence in Corrosion, King Fahd University of Petroleum and Minerals, Dhahran 31261, Saudi Arabia; mrahman@kfupm.edu.sa; Tel.: +96-6138607210

**Keywords:** polyurethane, ZnO, composite

## Abstract

A polyurethane (PU) is a multifunctional polymer prepared by using more than two types of monomers. The unique properties of PU come from monomers, thus broadening the applicability of PU in many different sectors. The properties can be further improved by using many nanoparticles. Different metal oxides as nanoparticles are also widely used in PU materials. ZnO is a widely used inorganic metal oxide nanoparticle for improving polymer properties. In this review article, the techniques to prepare a PU/ZnO composite are reviewed; the key protective properties, such as adhesive strength and self-healing, and applications of PU/ZnO composites are also highlighted. This review also highlights the PU/ZnO composite’s current challenges and future prospects, which will help to broaden the composite practical application by preparing environmentally friendly composites.

## 1. Introduction

**Figure 1:**
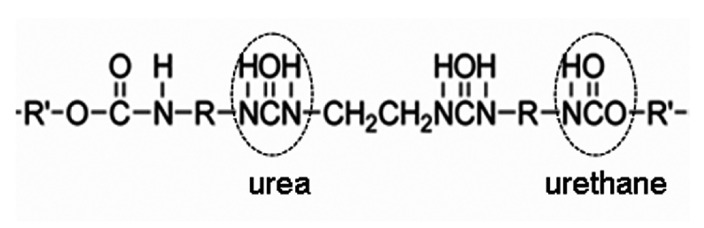
Urethane/urea group in polyurethane (PU).

A polyurethane (PU) material is considered to be a multifunctional polymer that consists mainly of urethane/urea groups (see Figure 1). During the Second World War, Otto Bayer, a German scientist, first synthesized PU. Over time, the PU has been improved by many research groups worldwide. Currently, the PU is widely used in many different applications. The main applications are as coatings, paints, adhesives, biomaterials and foams [1,2,3,4,5,6,7,8,9,10,11,12,13,14,15,16,17,18,19,20,21,22,23,24,25,26].

The main monomers of the PU are polyol, isocyanate and small-molecular-weight amine/diol. The varieties of monomer choices, which have unique properties, make it easy to target specific applications. The properties such as mechanical strength, thermal stability, barrier resistance and adhesive strength are tuned by using different polyol, isocyanate and amine/diol monomers and their contents [1,2,3,4,5]. The properties were further tuned by using different nanoparticles, such as clay, CNT, metal oxide, hydroxyapatite and graphene [19,20,21,22,23,24,25,26]. Usually, the properties are at an unsatisfactory level when using unbalanced amounts (very low or very high content) of monomers and nanoparticles [27]. At the early stage, mainly petroleum-based toxic monomers were used in PU preparation. Recently renewable-resourced monomers, as well as less-toxic monomers, are also being used. Therefore, the PU is considered to be a less-toxic material [1,2,18].

The addition of different metal oxides as a nanoparticle into PU can enhance the material properties and broaden their applications. Widely used metal oxides are ZnO, CeO_2_, CuO and TiO_2_ [26,28,29,30,31,32,33,34,35,36,37,38,39,40]. In the last two decades, a significant property improvement has been found by using the above metal oxides nanoparticles in the PU matrix. In particular, ZnO nanoparticle enhances the material properties and is being used in many different areas [31,32,33,34,35,36,37,38,39,40,41,42,43,44,45,46,47,48,49,50,51,52,53,54,55,56,57,58,59,60,61,62,63,64,65,66,67,68,69,70,71,72,73,74,75,76,77,78,79,80,81,82,83,84].

ZnO has unique properties, such as excellent photostability and chemical stability; furthermore, it has an electrochemical coupling coefficient and a broad range of radiation absorption [31,32,33,34,35]. Due to its low toxicity, biodegradability and biocompatibility, ZnO has been used with many polymers [81,82,83,84,85]. In many cases, ZnO was preferable to other metal oxides (such as CeO_2_, CuO and TiO_2_)_,_ because ZnO is comparatively very cost-effective, is free of surface water has and has an easy synthesis process [34,37,38,39,40,41,50,62,64,86].

**Figure 2:**
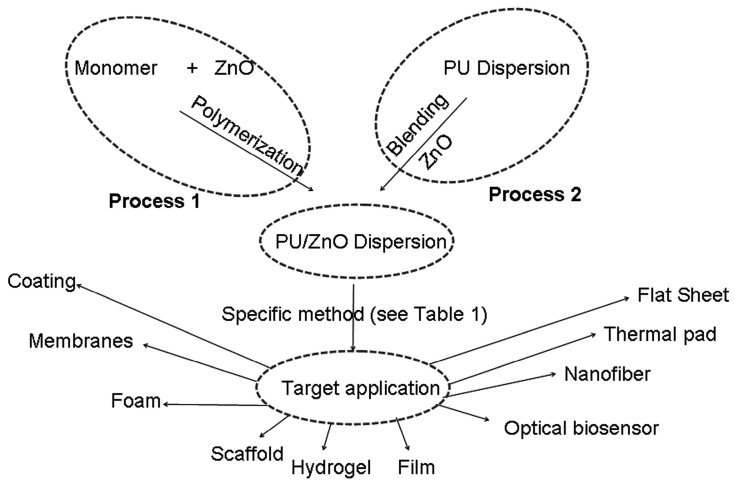
Preparation of PU/ZnO composite and their application.

Many articles published in the last two decades highlight the application of a PU/ZnO composite [31,32,33,34,35,36,37,38,39,40,41,42,43,44,45,46,47,48,49,50,51,52,53,54,55,56,57,58,59,60,61,62,63,64,65,66,67,68,69,70,71,72,73,74,75,76,77,78,79,80,81,82,83,84,85,86,87,88,89,90,91,92,93]. Research is still ongoing by many research groups, and their intention is to find solutions for new challenges, using PU/ZnO composites. However, there is no single review paper on the PU/ZnO composite. It is important to summarize the work and identify unexplored areas. The main highlight of this article is the review of the preparation method of the composite and their key protective properties, the application of the composite and the future prospects of the PU/ZnO composite (see Figure 2). All the published papers [31,32,33,34,35,36,37,38,39,40,41,42,43,44,45,46,47,48,49,50,51,52,53,54,55,56,57,58,59,60,61,62,63,64,65,66,67,68,69,70,71,72,73,74,75,76,77,78,79,80,81,82,83,84] mention that the physical, thermal and mechanical properties improve when using a proper ZnO content in the PU/ZnO composite; thus, the mentioned properties are not considered in this review article. However, the adhesion and self-healing properties of the PU/ZnO composite are reviewed, because both are important parameters of protective application, and without sufficient adhesive strength, it is not possible to use any composite for any kind of application or protection. Self-healing can act as a repairing tool during the service time. As there are separate papers on basic polyurethane [1,2,3] and ZnO [81,94,95,96,97,98], the present article also does not highlight PU monomers, different PU or ZnO synthesis processes.

## 2. Zinc Oxide Nanoparticles

ZnO is an n-type metal-oxide, which has the band gap 3.37 Ev; thus, it exhibits excellent semiconducting properties. Recently, ZnO is being widely used in optics, electronics, coating, elastomer, sun-screen and biomedical applications [97,98,99,100,101,102]. There are different methods to ZnO synthesis which are available in many reports [93,102]. The popular methods are the sol–gel process, hydrothermal synthesis, precipitation in water solution, precipitation from microemulsions, vapour deposition and mechanochemical processes. ZnO NPs have been reported in different morphologies, like the nanoflake, nanoflower, nanobelt, nanorod and nanowire with different sizes. The mean particle size varied from few nm to μm. The size and morphology of ZnO can be changed by tuning synthesis conditions and reactants [92]. Pholnak et al. tuned reaction time, temperature and mechanical forces during mixing the reactants to synthesize ZnO. They found the ZnO morphology changed with different temperatures. The ZnO was hexagonal prisms and hexagonal linked rods at 70 and 80 °C, respectively. The morphology changed from hexagonal linked rods to sword-like rods when they applied ultrasonic wave during the mixing of the reactants. They also showed that the mean particle size can be changed due to the concentration of reagents. The mean particle size decreased from 1.2 μm to 650 nm, using a higher concentration of C_6_H_12_N_4_ with Zn(NO_3_)_2_ [92]. In PU/ZnO composites’ preparation, both pristine and functionalized ZnO were used. The pristine ZnO was mainly from commercially available sources. In functionalized ZnO, the functionalization was done in different ways. It also depends on the attached functional group. However, the basic steps during functionalization were almost similar. Mostly the surface hydroxyl group of ZnO reacted with reactive group (mainly amine group or isocyanate group) to functionalize ZnO.

## 3. PU/ZnO Composite Dispersion Preparation

The PU/ZnO composite dispersion preparation is summarized in Figure 2. Either an in situ polymerization (Process 1) or blending (Process 2) is adopted to prepare PU/ZnO composite dispersion, which is then followed by different techniques to form various application formats (see Table 1).

### 3.1. In Situ Polymerization

**Scheme 1:**
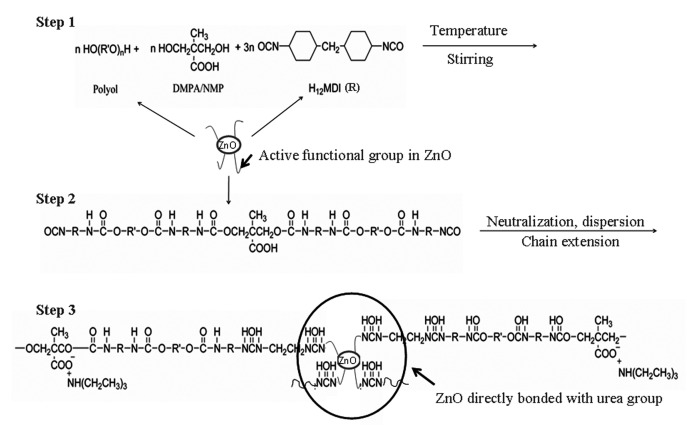
Typical PU/ZnO composite dispersion preparation using functionalized ZnO.

During in situ polymerization, steps such as prepolymer formation and chain extension are followed, similar to pristine PU dispersion preparation [19,20,21,22,23,24,25,26,27]. In situ polymerization is a popular synthesis process to prepare PU/ZnO dispersion [32,35,38,43,52,55,63,65,66,67,70,71,80,82,88], and it is almost similar to the preparation of pristine PU dispersion. During this type of polymerization, the polyol and diisocyanate monomers are charged together, to make an NCO-terminated prepolymer, which is followed by chain extension by using small-molecular-weight-based diol or amine. ZnO (pristine or functionalized) is mixed either monomer (polyol or isocyanate) or prepolymer (see Table 2, Table 3 and Table 4). Additional neutralization and water-dispersion steps are performed only for waterborne (WB) PU/ZnO dispersion [22,23,24]. In a typical procedure to prepare WBPU/ZnO [32,52], the PTMG, isophorone diisocyanate (IPDI) and 2,2-bis(hydroxymethyl) propionic acid (DMPA) in an NCO/OH molar ratio of 1.7 are mixed in a glass reactor, under a nitrogen atmosphere. The reaction continues until the NCO-terminated prepolymer is formed. ZnO is mixed with the prepolymer. Organic solvents such as acetone or 2-butanone are mixed with the reaction mixture to control the viscosity of the pre-polymer. Then, triethylamine (TEA) is added to neutralize the acid group of the prepolymer. The proper amount of water is added to the prepolymer that is dispersed in water. In the last step, all the free NCO groups of the prepolymer react to the amine group of ethylenediamine (EDA). This step mainly increases the molecular weight of the polymer. The organic solvent used is collected separately by a distillation process during the preparation of a WBPU dispersion [18]. If ZnO has any reactive groups (such as amine or hydroxyl) present, they react directly with isocyanate (see Scheme 1) [35]; otherwise, ZnO mainly interacts with PU by hydrogen bonding [32].

Completely different methods, such as (I) monomer mixing and bubble nucleation, (II) foam rising, (III) phase separation and cell opening, and (IV) foam formation, are used during the preparation of PU/ZnO composite foam [38,68].

### 3.2. Blending

**Scheme 2:**
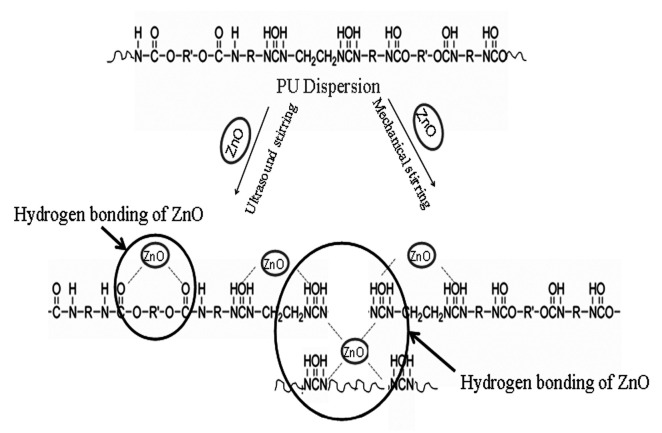
Typical PU/ZnO composite dispersion preparation by blending process.

Blending is a physical particle mixing process in polymer dispersion without any post-polymerization. Most of the reported [31,33,34,36,37,39,40,41,44,46,47,49,50,51,53,54,56,57,59,60,61,64,68,69,72,73,74,75,76,77,78,79,85,86,87,89,90,91,92,93] PU/ZnO composite dispersions are prepared by following this process (see Table 2, Table 3 and Table 4). In this process, the prepared PU dispersion (synthesized or collected from commercial grade) is blended with different ZnO contents, to prepare the target PU/ZnO composite (see Scheme 2). The defined ZnO content is mixed with ready PU dispersion, following the mechanical stirring or ultrasonicated technique mostly at room temperature. Most importantly, the mixing time with different ZnO content to achieve the homogeneous dispersion reported by different researchers varies [31,34,35,40,60,69,79].

### 3.3. PU/ZnO Composite Preparation for Different Application

The prepared PU/ZnO dispersion is used to make different composite film/coating/membrane for varieties application (see Table 1). The composite film/coating/membrane are formed by applying different methods, such as solution casting [31,34,36,37,51,91], dip coating [33,60,92], a Meyer rod method [76], spraying [40,46,61,79], spin coating [43,54,77,85], electrocoating [53,87], an automatic applicator method [78], a spiral applicator method [86], a freeze-extraction method [68], electrospinning [49], dipping [67], a solvent evaporation method [58], a hot press method [63,70], a high volume low pressure (HVLP) method [69], compression molding [48] and a drawdown method [59]. In most of the cases, the defined amount of dispersion poured into the substrate and kept an ambient condition to dry it completely. The dried coating further cured at moderate high temperature. The PU/ZnO composite properties highly depend on the mixed status of ZnO. The composite properties are improved only by a homogeneous mixing of ZnO. Otherwise, ZnO has detrimental effects on the composite properties. Most importantly, the optimum ZnO content to achieve target protective properties reported by different researchers varied, despite similar compositions and preparation processes [31,34,35,40,60,69,79].

## 4. PU/ZnO Composite Dispersion Stability

It is already proved that making a stable PU composite dispersion is challenging [23,25]. It depends on PU monomer, nanoparticle and mechanical forces [25]. The dispersion stability affected by both of nanoparticle-structure and -contents. The dispersion is stable up to certain nanoparticle contents. Polar-group-attached nanoparticle-based PU dispersion has higher stability [25]. The dispersion stability also varied with synthesis conditions. Although a lot of work has been done in PU/ZnO dispersion, only a few works mentioned their stability. In a stable composite dispersion, the particle remains in dispersion without any phase separation. The charges of particles make a repulsive force, which keeps the particles away from each other, to make the dispersion stable. In PU/ZnO, the dispersion was stable using both pristine and functionalized ZnO. The functionalized ZnO can increase the dispersion stability [60]. G. Christopher functionalized ZnO with oleic acid, the functionalized ZnO based PU dispersion was more stable that the pristine ZnO [60]. The oleic acid directly bonded with ZnO to avoid the aggregation of particles due to repulsive forces of particles; eventually, the dispersion was stable. The homogeneous distribution of ZnO is also important for a longer shelf life. The dispersion with functionalized ZnO was stable for six months, whereas the pristine ZnO based dispersion was stable only for 10 min.

## 5. Key Factors of PU/ZnO Composite for Protecting Application

The main application of PU/ZnO composite was protective purposes, especially for corrosion [31,34,35,40,60,69,79], fouling [37,72], UV-degradation [31,33,36,39,40,44,54,74,76,85,86,87,88,92,93] and bacterial [35,41,49,51,55,62,64,65,66,67,68,79,82,91,93]. There are few common criteria of materials to use as a protective material. The adhesion and self-healing properties are very important for any protective material. Moreover, the particle size of a nano particle also has a huge impact on polymer-composite properties.

### 5.1. Adhesion

Adhesion is an important parameter for any material used for coating on any substrate. Adhesion can be defined as the attachment of a material to a surface; usually, such a material is called an adhesive. Although there are natural adhesives, widely used adhesives are mainly synthetic polymer-based materials. Widely used polymer-based adhesives include rubber, polyacrylate, epoxy and polyurethane [1,2,3]. Usually, adhesion is quantified by strength, called adhesive strength. To evaluate the adhesive strength, it is necessary to detach the tested coating from the underlying metal substrate. In most cases, the adhesive strength is measured by a pull-off test. The general mechanism of improvement of adhesive strength is the increase in intermolecular forces between the substrate (mainly metal) and the adhesive. Recently, PU adhesive materials have been widely used due to not only their good adhesive strength but also their formulation flexibility and excellent weather resistance. The other advantage of PU adhesive materials is their flexible nature to bond many different substrates, such as fabrics, wood, rubber, plastics, leather and metals, making these adhesives applicable in the packing, transport, buildings and furniture industries. The preparation of PU adhesive material is also simple, consisting mainly of a reaction between polyol and diisocyanate. Most recent landmark advances in PU adhesives are environmentally friendly, solventless, biodegradable and renewable; all of these advances in PU make possible a broad range of applications. The adhesive strength of PU adhesives was improved by using appropriate monomers, crosslinkers and fillers [21,22,23,24,25,26,27]. Different nanoparticles and metal oxides have also been used as fillers to improve the adhesive strength of coatings. Among the different less-toxic metal oxides, ZnO was used in the PU coating. An improved adhesive strength was mostly recorded by using ZnO in the PU/ZnO coating. The ZnO content, adhesive strength and adhesion mechanism are summarized in Table 5. Steel substrates were mainly used to check the adhesive of PU/ZnO coatings. Both unmodified and modified ZnO were used, although the ZnO content varied widely from 0.1 to 6.0 wt.%. The adhesive strength also varied, and the adhesive strength increased with increasing ZnO content. The mentioned reasons for the adhesive strength are the chemical interaction between PU and the ZnO surface, reduced UV-degradation, hydrogen bonds and coordination bonds, and the ZnO reinforcement effect. The surface hydroxyl group of ZnO interacted with urethane, urea and ether groups (electronegative element), making a strong bond, and hence the adhesive strength increased. At exposed condition, the coating usually degraded by UV radiation. The degraded coating started to chain scission, which also help to passing electrolyte through the coating. Ultimately it shows negative effect on adhesive strength. As ZnO protect the UV-degradation, it also assumed that the chain scission is also opposed. Thus, ZnO protects the adhesive strength at exposed condition. Researchers have studied the effect of ZnO on the adhesive strength of PU/ZnO coatings during PU/ZnO composite coating for different practical applications, such as corrosion [40,52,79], antibacterial and antistatic properties [79,91], UV-degradation [87], anti-electrostatic properties [70], photopolymerization [71] and weather resistance [78]. Only Bravo et al. showed the effect of temperature on adhesive strength during PU/ZnO composite preparation. A higher adhesive strength was recorded by using a PU/ZnO composite prepared at a high temperature [40]. Other reviewed articles only examined the adhesive strength with different ZnO contents; in all cases, the adhesive strength increased with increasing ZnO content.

### 5.2. Self-Healing

Recent trends of polymer have been for it to regain its properties, especially the protective properties; this helps increase the shelf life of the material. Polymer with self-healing properties can be used for this purpose [99,100,101,102,103,104,105]. Such materials are getting much attention recently in the polymer and coating industry. This property is being utilized in many engineering materials. The main advantage of this property is automatic repairing after damage. In most of the cases, this damage cannot be seen with the naked eye, but this property helps to repair without external activity. The coating acts as a sustainable material. Usually polymers’ extrinsic and intrinsic properties are responsible for their self-healing properties. In the extrinsic case, the healing agent is pre-embedded intentionally during the coating preparation. In intrinsic coating, the coating possesses a repairing capacity through its internal chemical bond. Though both techniques are applicable for polymers, the intrinsic healing has more of an advantage, as this technique allows multiple time repairing [103,104,105]. Moreover, this technique allows good compatibility of bulk polymers, in which the interactions of functional groups triggered by heat, light and pH. Most importantly, the polymer chain has temporarily fast mobility to heal the damage [99]. In PU, the repairing mainly done by hydrogen bonds of multifunctional groups, such as urethane, urea, allophanate, etc. It has also been mentioned recently that reversible multi-crosslinks in PU can make multifunctional healing [58,99]. PU/ZnO coating also showed a self-healing property [58,100,101]. The inclusion of ZnO increases the hydrogen bond, as well as the shape memory, of the composite; hence, the self-healing property also increases [100,101]. The hydrogen bond can make a supramolecular structure, centered of ZnO. This structure helps to heal the scratched area [103,104,105].

### 5.3. ZnO Particle Size

The mean particle size of nanomaterial is important, especially for protective purposes of PU composite coatings. The protective properties, such as corrosion, UV-degradation and hydrophobicity, all are hugely affected by the different particle sizes of the same nanoparticles in a similar formulation. Based on the reviewed articles, the range of ZnO mean particle size was varied from 20 to 650 nm in PU/ZnO composite. Therefore, the used ZnO can be classified as macro- and nanoparticle. However, the mean particle size was below 100 nm mostly. Only few articles compared the effect of mean particle size of ZnO on their properties. Yang et al. [97] used ZnO to evaluate the effect of particle size of ZnO on a coating’s protective properties. The used mean particle size was 20 and 400 nm. The coating corrosion protection increased from using both ZnO particle sizes. It was also found that the coating corrosion protection rate was much higher from using the smaller size than the larger one. When the mean particle size is small, the particle has greater surface to absorb more polymers; hence, the corrosion resistance increased [97,98]. Moreover the smaller particle size can easily penetrate the coating’s micro-holes and, thus, oppose more strongly to pass the electrolyte. Ultimately, the corrosion resistance was further improved [97].

## 6. PU/ZnO Composite Applications

PU/ZnO composite has a wide range of application. ZnO itself has various protective properties. Additionally, ZnO functionalization with certain groups broadens PU/ZnO composite applications in different areas. Recent advances toward green and renewable resources of PU have also contributed to attracting industries, which has promoted an accelerated growth of new applications for PU/ZnO composite.

### 6.1. Antibacterial/Antimicrobial Application

Bacterial attack on different polymer material is a common issue. In a suitable environment, bacteria can grow on the material, thus making it a huge challenge to use that material. To protect the material from bacterial attack, different approaches have been followed. One of the useful ways is to enhance antibacterial properties by using different metal oxides nanoparticles with the proper material [84]. ZnO has also been used in PU/ZnO nanocomposites to enhance antibacterial properties [84]. It is common knowledge that a pristine PU material has negligible antibacterial properties [35,84]; however, with the inclusion of ZnO in the PU, the antibacterial properties improve significantly. Different PU/ZnO composites, such as coatings [35], foams [68], membranes [65,66], packaging films [64] and scaffolds [49], have been used for antibacterial purposes. The antibacterial properties of the PU/ZnO composite are summarized in Table 6.

**Scheme 3:**
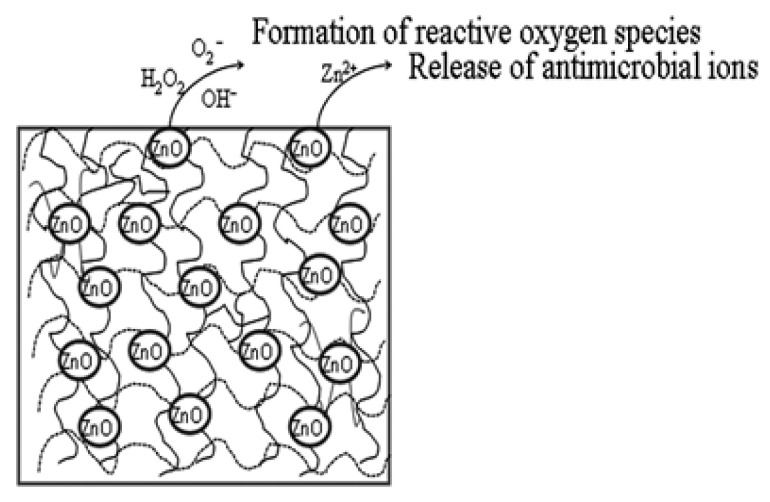
Mechanism of PU/ZnO antibacterial coating.

Although there are many reports [35,41,49,51,55,83] available on the antibacterial mechanism of ZnO, it is very difficult to find the exact antibacterial mechanism of the PU/ZnO composite. Notably, the antibacterial mechanism of the PU/ZnO composite has not yet been clearly evidenced. However, factors contributing to the antibacterial mechanism have been mentioned by different researchers (see Scheme 3) and are listed as follows:

1. The release of antimicrobial Zn^2+^ ion [55,64,79,82];
2. The antimicrobial property was due to ZnO [35,41,48,51,65,66,68];
3. It is the electrostatic interaction between the nanoparticles and microorganisms that ultimately kills the bacteria [49]; and
4. Reactive oxygen species are formed, which are also toxic to microorganisms [41,49,51,55,62,64,79].

Besides the microporous structure of the composite [82], the mechanical damage of the cell membrane by ZnO penetration also helps to enhance the antibacterial activity [55,79]. At least one of the mentioned factors should be available when using the PU/ZnO composite for an antibacterial purpose.

### 6.2. Anticorrosion Application

Corrosion is a natural phenomenon; most bare metals corrode quickly. Corrosion occurs as a result of electron transfer in a suitable corrosive environment. A metal was used as the anode to generate ions to initiate the corrosion process. Different methods involve inhibition of dopant anions, cathodic or anodic protection, barrier protection and shifting of electrochemical interfaces to protect the metal from corrosive environments [34,40,60]. To prolong the anticorrosion properties of metals, different organic coatings have been used on metals and their structures. PU is also used as an inhibitor, as well as a coating material. Different hydrophobic functional groups, such as siloxane and fluoro groups, have been attached to PU to improve its anticorrosion properties. To further improve the anticorrosion properties, different metal oxides as a nanoparticle have also been used in PU coatings. ZnO nanoparticle has also been considered to enhance the anticorrosion properties of PU. The corrosion protection mechanism and ZnO criteria of PU/ZnO nanocomposite coatings are summarized in Table 7. Commercially available PU is mainly used for this purpose. Different ZnO contents, from low to high content, are considered. Interestingly, the optimum ZnO content varies. As the commercial supplier did not disclose the monomers or their ratio, it is difficult to determine the reason for the differences in optimum ZnO content.

**Scheme 4:**
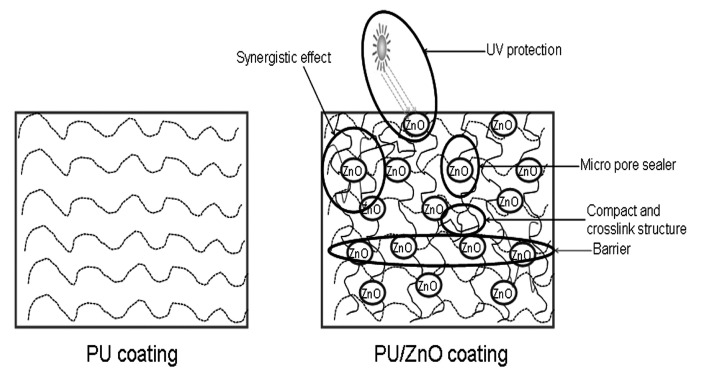
Mechanism of PU/ZnO anticorrosion coating.

The overall protection mechanism is summarized in Scheme 4. The anticorrosion enhancement can be ascribed to improved UV-degradation [31], barrier resistance by making compact and crosslinked structures [35], synergistic effects between the attached functional groups on ZnO [34], increased hydrophobicity [40], sealers [60] and chemical interactions [69].

Pristine ZnO has been used in PU/ZnO composite coatings by few researchers to protect the metal surface from corrosion [31,40,69,79]. It was reported that ZnO can act as an electrolyte barrier [31,79], UV absorber [31] and hydrophobic compound [40]. Functionalized ZnO also improved the corrosion resistance of the PU/ZnO composite coating [34,35,60].

### 6.3. Anti-UV-Degradation Application

Degradation over time is common to all kinds of polymer matrices. It is a partial decomposition of the matrix, and ultimately, it makes fragments with high molecular weight. Different factors that initiate degradation can be chemical (such as aggressive media and oxygen) as well as physical (such as heat, radiation and stress). The matrix degradation rate depends on the chemical structure, impurities (such as remaining catalyst and monomer), the exposed environment and the use of stabilizers. In an open-air atmosphere, most of the exposed PU material (mainly coating and film) is rapidly degraded by UV absorption. There are many different ways to enhance the anti-UV-degradation process. Metal oxide as nanoparticle use is one of the familiar methods to improve the anti-UV-degradation of the PU coating. ZnO has also been considered by many researchers [31,33,36,39,40,44,54,74,76,85,86,87,88,92,93]. A summary of their work is given in Table 8. They used different forms and contents of ZnO. The mechanism of anti-UV-degradation of the PU/ZnO compositeis improving its UV absorbing nature and the presence of synergistic effects.

### 6.4. Gas Separation Application

Polymeric membranes have been used in gas separation for almost 30 years. Although there are some other methods, such as pressure swing adsorption, amine scrubbing and cryogenic distillation, in gas separation applications, the main advantage of using polymeric membranes is their easy application, comparatively low cost and low energy consumption. Especially in the refinery industry, gas separation is crucial due to H_2_ purification, CO_2_ capture and hydrocarbon separation. There are published reports on using either PU or ZnO in membranes for gas separation, but only one article has been published using PU/ZnO composite membranes [56]. The authors showed that the permeability of N_2_, CO_2_ and CH_4_ depends on the ZnO content. The permeability increased with increasing ZnO content up to 0.50 wt.%, while the permeability decreased by using ZnO above 0.50 wt.%. Up to 0.50 wt.%, the ZnO was placed properly in the membrane, without creating barrier. Thus, the permeability increased. Above 0.50 wt.%, the excess amount of ZnO made a barrier, and ultimately the permeability decreased [56].

### 6.5. Medical/Biomaterial Application

PU material use in medical applications is common. A few researchers have also used PU/ZnOcomposite materials for this purpose [49,65,68,73]. The mechanical strength, biocompatibility and antibacterial properties of PU/ZnOcomposite materials are the main advantages for their use in medical applications. The main uses of PU/ZnOcomposite materials for medical and biomedical applications are hospital bedding, tubing, wound dressing, surgical drapes, injection equipment and implants.

### 6.6. Antistatic Application

Recently, the antistatic properties of materials and coatings are in demand because this property plays an important role in protecting a surface from static electricity. Generally, antistatic coatings are made by using metal oxides or nanoparticles. The PU/ZnO composite coating also exhibits good antistatic properties when ZnO is mixed homogeneously. The antistatic properties are also enhanced by using aniline groups during the preparation of the PU/ZnO composite coating [70,90].

### 6.7. Marine Antifouling Application

Marine applications have opened a new field for PU/ZnOcomposite materials [37]. Due to the banning of toxic tin-based compounds, new initiatives are in high demand in the marine paint and coating industry. A few published articles already prove that PU can be an option as an antifouling coating if the monomer and its contents are maintained properly. PU/ZnOcomposite coatings have also been used to protect the surface from the attachment of the fouler. Using a proper amount of ZnO in the PU/ZnOcomposite coating provides excellent fouling protection [37,72].

### 6.8. Electronic Device Application

The band gap (Eg=3.3 eV) and exciton binding energy (60 meV) of ZnO make it very attractive in electronic device applications, especially for memory arrays, microlasers, biosensors and chemical sensing. Only one work has been published using PU/ZnO composite material in this regard, and the PU/ZnO composite coating showed dielectric properties [43].

## 7. Future Prospects

All the reports reviewed here did not find any difficulties or challenges during the preparation of the various PU/ZnO composites. However, the use of large differences in ZnO content is quite confusing. In most cases, they do not mention the stability and shelf life of the solution after preparation. Only one article [60] mentioned the stability of PU/ZnO dispersion. The authors mentioned a very short storing time (only 10 min), using pristine ZnO. However, the storing time increased to six months by using functionalized ZnO. Counting 10 min storing is practically unacceptable to consider for a commercial and practical application. Surely, the other factors such as ZnO particle size, as well as PU monomer and their contents, may contribute to improve this limitation.

In addition, most of the applications are proved in the lab. Thus, they did not consider the real environment or simulated conditions. The lack of data in real environments may raise questions about the suitability of PU/ZnO composites in a real environment.

The availability of monomers and easy synthesis processes help to make a variety of PU/ZnO composites, which broadens their applications. Recent initiatives to use renewably resourced monomers of PU also open a new window of PU/ZnO composite materials due to environmental legislation. Another advantage of ZnO is its functionalization, which can alter the PU/ZnO chemistry to find new areas of application.

The majority of the PU/ZnO composites are used for anticorrosion, anti-UV-degradation and antibacterial coating purposes. In all cases, it is necessary to check the adhesive strength. Due to inadequate adhesive strength, the coating might not be useful for a long time. Unfortunately, only a few researchers have evaluated the adhesive strength of coatings. None of them considered any harsh conditions during their adhesive test. It is also important to consider harsh application conditions, which will help determine the real practical application value of the material. At the same time, a self-healing property was mostly not considered. It is an area that should be more focused, in order to overcome many coating failures.

Though large amounts of works are found on PU/ZnO composite material, there are a few areas that have not been considered both in synthesis and application. Only in situ polymerization and blending are considered during the preparation of PU/ZnO composite material. Not a single report was found based on controlled radical polymerization (CRP), such as RAFT or ATRP, and post-dispersion polymerization. These synthesis processes have been applied in pristine PU and have improved the matrix properties. By the CRP method, certain functional groups (e.g., azide groups) can be attached to the parent polymer chain, and the azide groups have various applications, due to their anticorrosion and antibacterial properties, which ultimately can broaden the application of PU/ZnO composite materials. In addition, the molecular weight can be controlled with the ATRP method; the ability to control molecular weight has a huge impact on certain properties. Post-polymerization can create a crosslinked structure, as well as provide the addition of certain functional groups in the parent chain, which may improve hydrophobicity. A large effort has also been noted for the synthesis of a PU matrix without the use of toxic diisocyanate. Unfortunately, no report has been found to synthesize PU/ZnO composite without isocyanate. If scientists are able to realize a PU/ZnO composite without isocyanate, the number of potential applications will dramatically increase.

Only two articles mention the use of CNTs and Ag nanoparticles in a PU/ZnO composite. Furthermore, there are many other nanomaterials available other than CNTs and Ag. By considering other nanoparticles (such as clay, Au nanoparticles and functionalized graphene),a new PU/ZnO composite material may be synthesized. These matrices can be used in anticorrosion, antifouling and anti-UV-degradation processes.

In the last decade, biodegradable matrices have become a prime interest. Unfortunately, most PU/ZnO composite studies overlook biodegradability. Only one report [64] was found on a biodegradable PU/ZnO composite material. More biodegradable PU/ZnO composite materials need to be explored, as biodegradable materials are now in high demand for human safety and environmental sustainability.

No report was found on the application of PU/ZnO composite material in implants. As PU/ZnO composite material has already been proven to be a good antibacterial and anticorrosive material, a PU/ZnO composite material may be used in implant applications.

PU is a very good biomaterial. Many studies have been performed on PU biomaterials. A PU/ZnO composite material may also be used as a healing material due to its antibacterial properties.

There are many reports available on lithium batteries using both PU and ZnO. However, not a single report was found using PU/ZnO composite materials in lithium battery applications.

For gas separation applications, only one article has been published using a PU/ZnO composite membrane. PU membranes are widely used for gas separation, and a very recent idea is the use of MOFs in gas separation applications. Therefore, MOFs with PU/ZnO composite membranes may be a very promising CO_2_ reduction application.

The PU/ZnO composite can be prepared in a more environmentally friendly way if scientists consider renewably resourced monomers and water as the main solvent. By considering the many different properties of the PU/ZnO composite, an increase in new applications can be explored in the near future.

## Figures and Tables

**Table 1:** Basic monomers, dispersion preparation methods and applications of PU/ZnO composite.

Application	Composite Dispersion		Preparation Process of Application	Reference
	Basic Monomers	Preparation		
Coating	Not mentioned, commercial grade	Blending	Solution casting	[31,34,36,37,51,91]
	Not mentioned, commercial grade	Blending	Dip coating	[33,60,92]
	Acrylic PU, commercial grade	Blending	Meyer rod	[76]
	Not mentioned, commercial grade	Blending	Spray	[40,46,61,79]
	Polyol, diisocyanate	In situ polymerization	Solution casting using square shaped applicator	[35]
	Polyol, diisocyanate	In situ polymerization	Spin coating	[43]
	Not mentioned, commercial grade	Blending	Spin coating	[54,77,85]
	Not mentioned, commercial grade	Blending	Electro coating	[53,87]
	Polyol, diisocyanate, pendant acid, 2-hydroxyethyl methacrylate	Blending	Automatic applicator	[78]
	Not mentioned, commercial grade	Blending	Spiral applicator	[86]
	Polyol, diisocyanate, pendant acid, triethylamine, ethylene diamine	Blending	Solution casting	[90]
Membranes	Not mentioned, commercial grade	Blending	Solution casting	[56,75]
	Polyol, pendant acid, diisocyanate, butane diol (as a chain extender)	In situ polymerization	Precipitation process using warm distilled water	[65,66]
Foam	Polyol, diisocyanate	In situ polymerization	Water blown in an open mold	[38]
	Polyol, diisocyanate, butane diol	Blending	Freeze-extraction	[68]
Scaffold	Not mentioned, commercial grade	Blending	Electrospinning	[49]
Hydrogel	Polyol, diisocyanate	In situ polymerization	Dipping	[67]
Film	Polyol, pendant acid, diisocyanate, tertiary amine (as a base), diamine (as a chain extender)	In situ polymerization	Solvent evaporation	[32,52,55,88]
	Polyol, diisocyanate, butanediol	In situ polymerization	Solution casting using a ruler	[80]
	Polyol, pendant acid, diisocyanate, 2-hydroxyethyl acrylate, 2-hydroxyethyl methacrylate pentaerythritol triacrylate, 2-hydroxypropyl acrylate, hydroxypropyl methacrylate, 4-methoxyphenol	In situ polymerization	Solvent evaporation	[71]
	Polyol, diisocyanate, methylene-bis-ortho-chloroaniline	In situ polymerization	Hot press	[63,70]
	Diisocyanate, N, N-bis(2-hydroxyethyl)isonicotinamide	In situ polymerization	Heat treated at muffle furnace	[82]
	Polyol, diisocyanate	Blending	Solution casting	[64,74]
	Not mentioned, commercial grade	Blending	High volume low pressure (HVLP)	[69]
	Not mentioned, commercial grade	Blending	Solution casting	[39,41,47,72,73]
	Commercial grade, polyol diisocyanate, diamine	Solution blending	Solution casting	[50]
	Polyester PU, commercial grade	Blending	Spin coating method, solution casting	[44]
	Not mentioned, commercial grade	Melt processing	Compression moulding	[48]
	Not mentioned, commercial grade	Solution blending	Drawdown	[59]
Optical biosensor	Not mentioned, commercial grade	Spread over base PU	ZnO dried on polymerized PU	[45]
Nanofiber	Not mentioned, commercial grade	Blending	Electrospinning	[57,89,93]
Thermal pad	Polyol, diisocyanate, furfuryl alcohol, bismaleimide	ZnO/stainless steel (SS) mesh and PU prepared separately; PU solution poured on ZnO/stainless steel (SS) mesh	Solvent evaporation	[58]
Flat Sheet	Not mentioned, commercial grade	PU sheet was immersed in ZnO solution	Dipping process	[62]

**Table 2:** Mixing of pristine ZnO during PU/ZnO dispersion preparation.

ZnO Premixed with Solvent		Mixing Stage of ZnO				Reference
Yes	No	Mixed with Ready PU	Mixed In Situ Polymerization			
			Polyol	Isocyanate	Prepolymer	
…	✓	✓	…	…	…	[31,48,51,54,59,60,61,65,68,69,78,79,85,86,89,91,92,93]
…	✓	…	…	…	✓	[32,52,82]
✓	…	…	…	…	✓	[43,50]
…	✓	…	✓	…	…	[38,63,70,80]
✓	…	✓	…	…	…	[39,40,45,46,47,53,56,62,66,87]

**Table 3:** Mixing of pristine ZnO along with other nano particle/metal oxide/polymer during PU/ZnO dispersion preparation.

Nano Particle/Metal Oxide/Polymer	ZnO Premixed with Solvent		Mixing Stage of ZnO				Ref.
	Yes	No	Mixed with Ready PU	Mixed In Situ Polymerization			
				Polyol	Isocyanate	Prepolymer	
*f*MWCNT	✓	…	✓	…	…	…	[49]
Graphene aerogel	…	✓	…	✓	…	…	[67]
Cellulose-acetate	…	✓	✓	…	…	…	[75]
Polylactide	...	✓	✓	…	…	…	[73]
Chitosan	…	✓	✓	…	…	…	[64]
stainless steel mesh	…	✓	✓	…	…	…	[58]
CdO	✓	…	✓	…	…	…	[57]

**Table 4:** Mixing of functionalized ZnO during PU/ZnO dispersion preparation.

Attached Functional Group in Modified ZnO	Other Material with ZnO	*f*-ZnO Premixed with Other Solvent		Mixing Stage of ZnO				Ref.
		Yes	No	Ready PU	Mixed In Situ Polymerization			
					Polyol	Isocyanate	Prepolymer	
3-aminopropyltriethoxysilane	..	..	✓	✓	..	..	..	[33]
Poly(o-toluidine)	..	✓	..	✓	..	..	..	[34]
Amine	..	..	✓	..	✓	..	..	[35]
Lignin	..	✓	..	✓	..	..	..	[36]
Chitosan	..		✓	✓	..	..	..	[37]
Aminopropyltriethoxysilane, activated stearic acid and carbonyldiimidazole	..	✓	..	✓	..	..	..	[39]
Magnesium	..	..	✓	✓	..	..	..	[41]
CeO_2_	..	..	✓	✓	..	..	..	[44]
2-aminoethyl-3-aminopropyltrimethoxysilane	..	..	✓	✓	..	..	..	[54]
3-aminopropyltriethoxysilane	..	..	✓	..	..	✓	..	[55]
Oleic acid	..	..	✓	✓	..	..	..	[60]
Hydroxy acrylate and toluene diisocyanate	..	..	✓	..	..	..	✓	[71]
Polyaniline	..	..	✓	✓	..	..	..	[72]
3-(Trimethoxysilyl)propyl methacrylate	..	..	✓	..	..	..	✓	[74]
3-aminopropyltrimethoxysilane	Corundum, SiO_2_	..	✓	✓	..	..	..	[76]
Ag	..	..	✓	✓	..	..	..	[77]
Amphiphilic PU	..	✓	..	..	..	..	✓	[88]
Polyaniline	Copper	..	✓	✓	..	..	..	[90]

**Table 5:** Adhesion of PU/ZnO composite materials.

ZnO			Failure Type	Testing Method	Thickness	Substrate	Mechanism	Ref.
Nature	Used Content	Adhesive Strength (MPa)						
Pristine	2.0 wt.% 6.0 wt.%	3.8 4.7	Not mentioned	Pull-off test	20 μm	AISI 1018 carbon steel (CS)	(1) Chemical interaction between PU and ZnO surface.(2) Reduced UV-degradation	[40]
Pristine	0 0.25 wt.% 0.50 wt.% 0.75 wt.% 1.00 wt.%	7.1 8.4 11.4 12.2 15.4	Not mentioned	Pull-off test	Not mentioned	Carbon steel	(1) Hydrogen bond which increased with increasing ZnO content.(2) Coordination bond between metal ions and free CO groups	[52]
Hydroxy acrylate and toluene diisocyanate functionalized	0.2 wt.% 0.4 wt.% 0.6 wt.% 0.8 wt.% 1.00 wt.%	1 1 0 0 0	Not mentioned	GB/T 1720–1979	Not mentioned	Not mentioned	Not explained	[71]
Pristine	0% 1.0 wt.% 3.0 wt.% 5.0 wt.%	4.5 7.1 7.6 8.0	Not mentioned	Pull-off test	Not mentioned	Glass plate	Not explained	[78]
Pristine	0% 0.1 wt.% 0.5 wt.% 1.0 wt.% 1.5 wt.% 2.0 wt.%	4 4 6 7 8 8	Not mentioned	Pull-off test	Not mentioned	Steel	ZnO reinforcement effect	[79]
Polyaniline functionalized	WBPU WBPU-PANI (5.0 wt.%) WBPU-Cu-ZnO (5.0 wt.%) WBPU-P-Cu-ZnO (5.0 wt.%)	7.10 7.20 7.40 7.70	100% Adhesion 25% Cohesion 40% Cohesion 80% Cohesion	Pull-off test	Not mentioned	steel	Polar and hydrophilic nature improve the adhesive strength	[90]

**Table 6:** Antibacterial properties of PU/ZnO composite.

PU/ZnO Composite	Attached Functional Group with ZnO	Antibacterial Test	ZnO Content	Antibacterial Activity						Ref.
				*E. coli*	*K. pneumonia*	*S. ureus*	*B. subtilis*	*A. niger*	*A. brasiliensis*	
Hybrid composite coatings	3-aminopropyltrimethoxysilane	Luria–Bertani agar medium	1.0 wt.% 1.5 wt.% 2.0 wt.%	+ ++ +++	++ + ++	+ ++ +++	− − −	N.D N.D +++	… … …	[35]
Composite film	Magnesium-doped	MacConkey agar	1.0 wt.% 2.5 wt.% 5.0 wt.%	67.6% 73.5% 93.3% (Antibacterial rate)	… … …	… … …	… … …	… … …	… … …	[41]
Hybrid Scaffold	MWCNT	Tryptic soy broth medium	0.2 0.4 wt.%	148% (Inhibition zone)	…	158% (Inhibition zone)	…	…	…	[49]
Coating	…	Agar dilution method	1.0 2.0 3.0 4.0	20 26 71 90	… … … …	… … … …	0 18 61 84	… … … …	… … … …	[51]
composite	3-aminopropyltriethoxysilane	Agar plate	0.5 wt.% 1.0 wt.% 1.5 wt.% 2.0 wt.% 4.0 wt.%	42 12 7 3 0 (survival ratio%)	… … … … …	70 42 25 8 0 (survival ratio%)	… … … … …	… … … … …	… … … … …	[55]
Bactericidal coating sheet	Crystal violet	MacConkey agar Mannitol Salt agar	Not mentioned	+++	…	+++	…	…	…	[62]
Packaging film	Chitosan	Agar plate	1.0% 3.0% 5.0%	+ ++ +++	… … …	+ ++ +++	… … …	… … …	… … …	[64]
Membranes	…	Sabouraud agar	5.0 wt.% 10.0 wt.%	…	…	…	…	…	+ ++	[65]
Membranes	…	Minimum inhibitory concentration method (cell viability)	2.0 wt.% 4.0 wt.% 6.0 wt.% 8.0 wt.%	+ ++ +++ ++++	… … … …	… … … …	… … … …	… … … …	… … … …	[66]
Composite foam	…	Mueller Hinton Agar	10.0 wt.%	10^3^ CFU (colony forming unit) reduction	…	10^3^ CFU reduction	…	…	…	[68]
Coating	…	Well diffusion	0.1 wt.% 0.5 wt.% 1.0 wt.% 1.5 wt.% 2.0 wt.%	15% 40% 65% 80% 100% (Inhibition)	… … … … …	… … … … …	20% 50% 75% 90% 100% (Inhibition)	… … … … …	… … … … …	[79]
Hybrid materials	…	Agar plate	Not mentioned	✓ +++	…	…	…	…	…	[82]
Varnished coating	…	Counted number of colonies	0.4 wt.% 0.7 wt.%	✓ 22 ✓ 16 ✓ (inhibit growth)	… …	8 (85%) 2 (95%) (inhibit growth)	… …	… …	… …	[91]
Nano fiber	…		1.0 5.0	✓ … ✓ …	60.0 98.7	99.9 99.9	… …	… …	… …	[93]

“+” = it has antibacterial activity, “−” = it has no antibacterial activity, N.D. = it has not determined.

**Table 7:** Corrosion properties of PU/ZnO composite.

ZnO			PU Monomer	E_corr_(mV)(at Optimum Content)	Substrate	Protection Mechanism	Ref.
Nature	Used Content	Optimum Content					
Pristine	0.1 wt.%, 1.0 wt.%	0.1 wt.%	Not mentioned, commercial grade	…	Mild steel	(1) Improved barrier resistance and (2) reduced UV-degradation	[31]
Poly(o-toluidine) functionalized	7.0 wt.%, 14.0 wt.%	7.0 wt.%	Not mentioned, commercial grade	−669.5	Carbon steel	(1) Passive layer formation, (2) synergistic effects of poly(o-Toluidine) and (3) coating porosity reduced by addition of ZnO	[34]
Amine functionalized	1.0 wt.%, 2.0 wt.%	2.0 wt.%	Polyol, diisocyanate	…	Mild steel	(1) Crosslinked structures formation, which ultimately improved barrier resistance	[35]
Pristine	2.0 wt.%, 4.0 wt.%, 6.0 wt.%	6.0 wt.%	Not mentioned, commercial grade	−692	AISI 1018 carbon steel	(1) Improved hydrophobicity and (2) compactness of the coatings	[40]
Oleic acid functionalized	0.1 wt.%, 0.3 wt.%	0.3 wt.%	Not mentioned, commercial grade	−678	Mild steel	(1) Filler acted to seal coatings pores and (2) improve the barrier protection	[60]
Pristine	1.0 wt.%, 3.0 wt.%, 5.0 wt.%	1.0 wt.%, 3.0 wt.%	Not mentioned, commercial grade	…	Low carbon steel	(1) Chemical interaction between ZnO and polyurethane	[69]
Pristine	0.1 wt.%, 2.0 wt.%	2.0 wt.%	Not mentioned, commercial grade	…	Steel	(1) Improve barrier resistance	[79]

**Table 8:** Anti-UV-degradation properties of PU/ZnO composites.

ZnO Nature	Content	Optimum Content	Lab Test for UV Resistance	Protection Mechanism	Ref.
Pristine ZnO	0.1–1.0%	0.1%	Salt Spray (ASTM B117),Humidity (ASTM D2247) and UV weathering (ASTM G53)	ZnO wide band gap (3.37 eV) and large excitation binding energy (60 meV), which ultimately absorb the UV light	[31]
Surface-modified hexagonal ZnO	5, 10, 20 wt.%	Not mentioned	UV absorption	ZnO wide band gap	[33]
Quaternized alkali lignin/ZnO	0.5, 1.0, 1.5 wt.%	1.0 wt.%	UV lamp	Synergistic effect	[36]
Aminopropyltriethoxysilane, stearic acid and N,N-carbonyldiimidazole	0.5, 1, 2, and 3 wt.%	2.0 wt.%	UV–vis spectrophotometer(GBC Cintra 10e, Australia)	Not explained	[39]
Pristine ZnO	2, 4, 6 wt.%	6 wt.%	UV–vis analysis	ZnO wide band gap and large excitation binding energy	[40]
Silane modified ZnO	Not mentioned	Not mentioned	self-designed comprehensive UV/ozone aging test device	This may be attributed to the composite UV absorbent which could effectively shield or absorb ultraviolet light and thus inhibit or reduce the rate of oxidation reaction of the incremental macromolecular and postpone the degradation and aging process of the PU material.	[44]
2-aminoethyl-3-aminopropyl-trimethoxysilane	0.1, 0.5 wt.%	0.1 wt.%	QUV test	UV blocking of modified ZnO	[54]
3-(trimethoxysilyl)propyl methacrylate modified ZnO	0.3, 0.9, 1.5, 3.0 g	Not mentioned	UV Spectrometer test	UV shielded by ZnO	[74]
(3-aminopropyl)trimethoxysilane and corundum nanoparticle	2 and 6 wt.%	2 wt.%	Accelerated weathering test	Synergistic effect	[76]
Pristine ZnO	1, 2 and 5 wt.%	Not mentioned	High UV-radiant laboratory exposure	UV shielded by ZnO;however, the ZnO can accelerate or reduce the UV-degradation, depending on the coating formulation, the particle size of ZnO and exposure conditions	[85]
Pristine ZnO	2 and 4%	Not mentioned	Natural and accelerated weathering test	UV absorbed in ZnO	[86]
Pristine ZnO	Not mentioned	Not mentioned	Accelerated weathering test	UV absorbed in ZnO	[87]
Amphiphilic PU	0.1, 0.3, 0.4 and 0.5 wt.%	0.3 wt.%	UV/VIS spectrophotometer	UV absorbed in ZnO	[88]
Pristine ZnO	5 and 15 wt.%	15 wt.%	UV/VIS spectrophotometer	UV absorbed in ZnO	[92]
Pristine ZnO	1 wt.%	Not mentioned	UV/VIS/NIR spectrophotometer	UV blocked by ZnO	[93]
